# A Printed and Flexible NO_2_ Sensor Based on a Solid Polymer Electrolyte

**DOI:** 10.3389/fchem.2019.00286

**Published:** 2019-04-26

**Authors:** Ru-bai Luo, Hai-bin Li, Bin Du, Shi-sheng Zhou, Yu-heng Chen

**Affiliations:** ^1^School of Print Packaging and Digital Media, Xi'an University of Technology, Xi'an, China; ^2^Shaanxi Collaborative Innovation Center of Green Intelligent Printing and Packaging, Xi'an University of Technology, Xi'an, China

**Keywords:** solid polymer electrolyte, gas sensor, screen printing, carbon electrode, double-layered composite structure

## Abstract

Solid polymer electrolyte (SPE) is an important part of printed electrochemical gas sensors and are of value to electrochemical sensors. Here, a new type of SPE was prepared by dissolving a poly-vinylidene fluoride (PVDF) matrix in a 1-methyl-2-pyrrolidone (NMP) to immobilize 1-ethyl-3-methylimidazolium tetrafluoroborate ([EMIM] [BF_4_]), which was then used in a new electrochemical amperometric nitrogen dioxide sensor. The SPE was coated on a single electrode and attached to the electrode to construct a simple two-layer structure. Nitrogen dioxide in the air was reduced on the working electrode at a bias voltage of −500 V. We controlled the components and process parameters separately for control experiments. The results show that the SPE based on [EMIM] [BF_4_], NMP, and PVDF coated on the electrode at a thickness of 1.25 mm with a 1:1:4 weight ratio under heat treatment conditions of 80°C for 2 min has the best sensitivity. The FTIR and XPS results indicated that SPE is prepared via physical miscibility. The SEM and XRD results showed that the sensitivity of the sensor is strongly dependent on the interconnected pore structure in SPE, and the pore structure is related to the synthesis ratio, morphology, and heat treatment mode of SPE. Moreover, the sensor sensitivity has a certain relationship with SPE conductivity. The reaction principle and cycle performance of the sensor were also studied.

## Introduction

Air pollution is a major problem (Mattana and Briand, 2016) leading to the development and miniaturization of gas sensors (Li et al., 2016). Printed gas sensors have attracted increasing attention due to their low cost, small size, and ease of manufacture (Zhao et al., 2011). Solid polymer electrolyte (SPE) is widely used in sensor manufacturing. Crowley et al. reported a fully printed SPE-based gas sensor to detect hydrogen sulfide with a detection limit of 2.5 ppm at 10–100 ppm. It had a linear relationship between current and concentration in the 100-ppm region (Crowley et al., 2013; Sarfraz et al., 2013). However, printed gas sensors based on SPE often have defects such as poor stability, low selectivity, complex sensitivity mechanisms, and short lifetimes (Korotcenkov and Cho, 2011). Therefore, the development of a SPE that satisfies better stability and has a simple reaction mechanism is an important hot spot for current and future researchers. For example, Petr Kuberský et al. fabricated an electrochemical gas sensor based on a SPE on a flexible substrate through a complete screen-printing technique that had no metal electrode while also being inexpensive and easily mass produced (Kuberský et al., 2014).

Room temperature ionic liquids consist entirely of ions that possess several properties including negligible vapor pressure, wide potential windows, high thermal stability, and good intrinsic conductivity. This makes them attractive alternative electrochemical solvents and potentially advantageous in the development of stable and robust gas sensors (Buzzeo et al., 2004). To date, several ionic liquids have been widely used for the preparation of SPE. Electrochemical sensors based on different ionic liquids have been used to detect nitrogen dioxide (Silvester, 2011), ammonia (Silvester, 2011; Carter et al., 2016; Sekhar and Kysar, 2017), oxygen (Zevenbergen et al., 2011; Toniolo et al., 2012), ozone (Bidikoudi et al., 2014), and ethylene (Li and Compton, 2015). Nádherná et al. used direct radical polymerization of a mixture of room temperature ionic liquid to detect NO_2_ at the platinum electrode (Kuberský et al., 2015). However, gas sensors based on SPE have a three-layer structure (Millet et al., 1996; Nádherná et al., 2011; Vonau et al., 2012; Manjunatha et al., 2014; Goto et al., 2015; Hodgson et al., 2015; Xie et al., 2015), and the fabrication process of this electrode-SPE-electrode composite structure is complicated. In addition, it is necessary to consider the surface bonding ability of the SPE and the double-layer electrode, which is often a constraint of large-scale mass production.

Based on this background, a new type of SPE was prepared from the synthesis of 1-ethyl-3-methylimidazolium tetrafluoroborate ([EMIM] [BF_4_]), 1-methyl-2-pyrrolidone (NMP), and poly-vinylidene fluoride (PVDF). [EMIM] [BF_4_] is a room-temperature ionic liquid with strong ions that make the liquid highly stable, and it becomes an ideal medium for gas detection. The application of ([EMIM] [BF_4_]) in gas sensors can improve the detection limits and increase gas solubility and create wider potential windows (Rogers et al., 2010). The SPE and electrode formed a simple two-layer sensing structure. The results showed that the structure of the gas sensor can detect NO_2_. The effect of the proportion of SPE and the control of the synthesis process parameters on the electrochemical performance of the gas sensor was studied to identify the best parameters. The microstructure, molecular properties, and material composition of different SPEs were also studied by scanning electron microscopy (SEM), Fourier transform infrared (FT-IR), X-ray diffraction (XRD), and X-ray photoelectron spectroscopy (XPS). The experiments confirmed the fabrication of a low-cost, fully-printed, and flexible gas sensor on a polyethylene terephthalate (PET) substrate.

## Experimental

### Materials and Characterizations

The 1-ethyl-3-methylimidazolium tetrafluoroborate ([EMIM] [BF_4_], C_6_H_11_BF_4_N, 99 wt%) and poly-vinylidene fluoride (PVDF, -(CH_2_CF_2_)n-, 98 wt%) were purchased from Love Pure Biological Technology (Shanghai, China) and used as received. The 1-methyl-2-pyrrolidone (NMP, C_5_H_9_NO, 99 wt%) was bought from Wuxi Yatai United Chemical (Wuxi, Jiangsu, China) and used as received. The morphology and cross-sectional microstructure of the SPE samples were investigated by field emission scanning electron microscopy (SEM, JEM3010, JEOL, Japan).

Fourier transform infrared (FT-IR) study was performed on a Fourier transform infrared spectrometer (IR, prestige-21/FTIR-8400S, Japan) in a KBr pellet. The resolution was 0.2 cm^−1^, the number of scans was 16 s^−1^, and the scan range was from 400 to 4,000 cm^−1^. X-ray photoelectron spectroscopy (XPS, AXISULTRA, Kratos, UK) was recorded to study the chemical state of the molecules presented at the SPE by using Monochromatic Al Kα (1,486.71 V) line at a power of 100 W (10 mA, 10 KV) with the vacuum of about 10^−8^ Torr. X-ray diffraction (XRD) was performed by using a D/MAX-2550 diffractometer (Rigaku International Co., Japan). The conductivity of the SPE were measured by a four-point probe resistivity measurement system (NAPSON Corporation, RT-70V/RG-5). The electrochemical studies used a potentiometer (PS-12, from Hebei Huachuang Instrument Factory, Jiangsu, China) at a best bias voltage of −500 V.

### Electrode Preparation

The interdigitated electrode was prepared by screen printing with carbon paste conductive ink. In a typical manufacturing process, the technological parameters of the JB-45CA screen printing machine (from Zhejiang Jinbao Machinery Co., Ltd. China) were set, the scraper pressure was 100 N/m, the scraper angle was 75°, and the scraper speed was 60 m/min. The carbon paste conductive ink was then evenly applied to one end of the printing plate, and the ink was transferred onto a PET film. The screen-printing plate had a screen number of 350 mesh/inch (1 inch = 0.0254 m) and had been exposed. Finally, the printed electrodes were vacuum dried at 120°C for 30 min.

### Synthesis of SPE

The SPE is composed of [EMIM] [BF_4_], PVDF, and NMP. Poly-vinylidene fluoride is the matrix and NMP is the solvent of PVDF. The ionic liquid determines the viscosity of the solid electrolyte on PET film and the structure of the SPE layer. Gregorio and Borges (2008) described the effect of different temperatures on the crystalline phase of PVDF dissolved in NMP. Therefore, we studied the effect of temperature and blending ratio on the morphology and porosity of SPE. Under different crystallization conditions, we also explored the influence of the SPE coating thickness on sensor gas sensitivity. Here, we synthesized several groups of SPE with different mixing ratios and different processing conditions (Table 1). Samples S1–S4 represent four weight ratios of [EMIM] [BF_4_], PVDF, and NMP. Samples S5–S9 represent SPE with different heat treatment conditions at optimum ratios. Samples S10–S14 represent the different thicknesses of the SPE coated on the interdigitated electrodes under optimum mixing and heat treatment conditions. We coated this via the equal displacement method. To obtain a uniform SPE, PVDF was gradually added to the mixed liquid in a CJJ78-1 magnetic heating stirrer (from Jintan Dadi Automation Instrument Factory, Wuxi, Zhejiang, China) over 20 min and stirred with a stirring capsule. The SPE was transferred to a D2F-1B vacuum drying oven (Shanghai Keheng Industrial Development Co., Ltd., China) until the SPE was transparent. This was then applied to uncooled SPE on the PET film substrate with interdigital electrode to completely cover the fork. Finally, the sample was held at a stable condition for 24 h under laboratory conditions, and the hydrophobic membrane was coated on the sample with a hot laminating machine.

**Table 1:** SPE parameters.

**Sample**	**Ratio**	**Temperature (^**°**^C)**	**Teat treatment time (min)**	**Thickness (mm)**
S1	1:1:3	80	2	1.25
S2	1:1:4	80	2	1.25
S3	1:1:5	80	2	1.25
S4	1:1:6	80	2	1.25
S5	1:1:4	80	2	1.25
S6	1:1:4	80	5	1.25
S7	1:1:4	80	8	1.25
S8	1:1:4	120	2	1.25
S9	1:1:4	160	2	1.25
S10	1:1:4	80	2	0.75
S11	1:1:4	80	2	1.00
S12	1:1:4	80	2	1.25
S13	1:1:4	80	2	1.50
S14	1:1:4	80	2	1.60

### Measurement Setup

The gas test system for the measurement of sensor characteristics consists of two gas tanks (the first filled with N_2_ and the second filled with NO_2_), exhaust gas collection devices, two flow controllers, and a sealed glass test room equipped with a potentiograph. The characteristics of all sensors were measured under identical conditions (unless otherwise stated: 20 ± 5°C, 50% RH). A potentiostat with stable potential was used to provide a stable voltage for the sensor. To prevent the influence of interfering substances from passing through a sufficient amount of N_2_ to exclude the test room air and then pass in the quantitative NO_2_, the required NO_2_ concentration is quantitatively controlled by the flow controller for the two gas tanks. The output analog voltage levels from each potentiostat were sampled every other second with two four-channel 24-bit analog to digital converters (ADC; ADS1256, Kangwei Electronics, China), and data were subsequently transferred to a coordinator connected to the computer via wireless sensor data transmission technology. At the end of the sampling, the gas remaining after the reaction was collected and processed through an exhaust gas recovery device.

## Results and Discussion

### Sensor Construction

**Figure 1:**
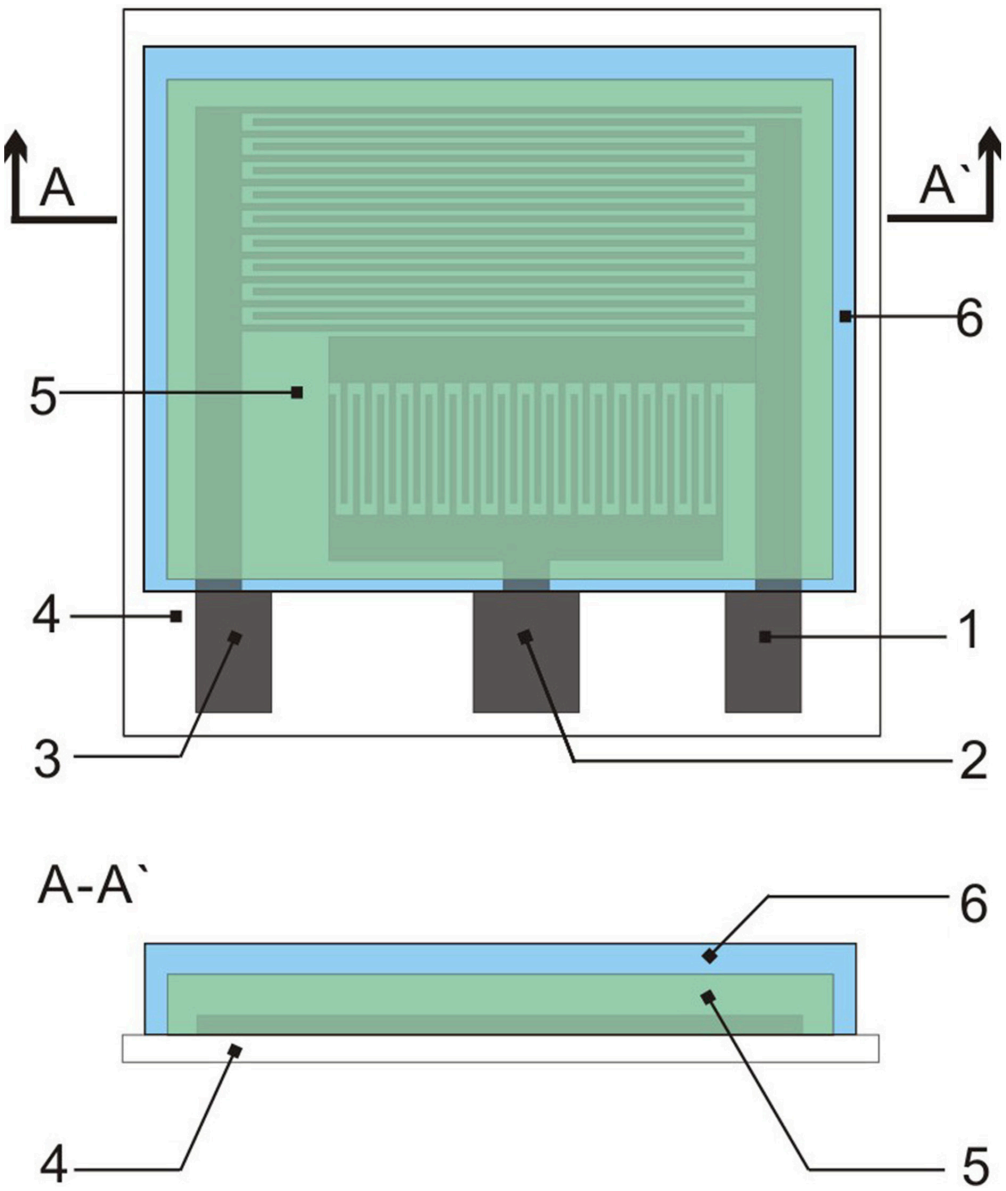
Topology of the sensor: (1) Indicator electrode; (2) Reference electrode; (3) Auxiliary electrode; (4) PET film; (5) SPE; and (6) Hydrophobic membrane.

**Figure 2:**
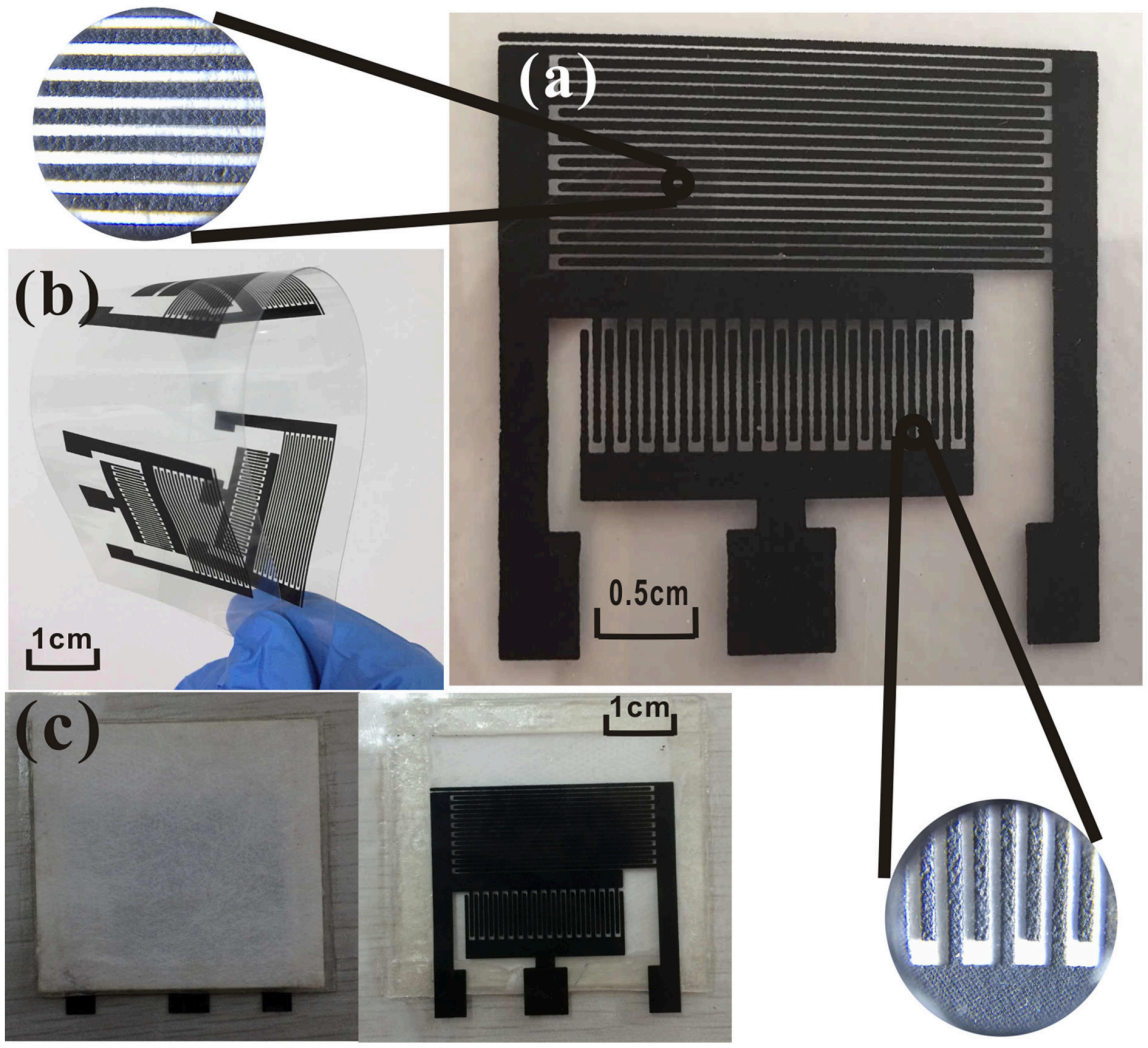
**(a)** Interdigitated electrode; **(b)** curved electrode; **(c)** NO_2_ sensor.

The gas sensor has three patterns on the PET film pair (Figure 1). The sensor platform (Figure 2c) is prepared on a PET film substrate (4 × 4 cm). The three-electrode system is composed of a working electrode, a reference electrode, and an auxiliary electrode, which has better potential stability and reproducibility than the two-electrode system (Ianeselli et al., 2014; Wardak, 2014). The electrode is closely attached to the SPE and forms the basic part of the sensor. We used a three-electrode system with interdigitated structures (Figure 2a) for capturing more components to be tested. This also saved samples, increased detection signals, and confirmed the distribution of the measured objects. Figure 2b shows that screen-printing technology allows the electrode to have a very clear grain, good stability, and good ductility in the bent state. The SPE covers most of the electrode area and only the three ports of the electrode are not attached. The hydrophobic breather membrane completely covers the SPE and is not in contact with the SPE.

### FT-IR Spectral Analysis of SPE

**Figure 3:**
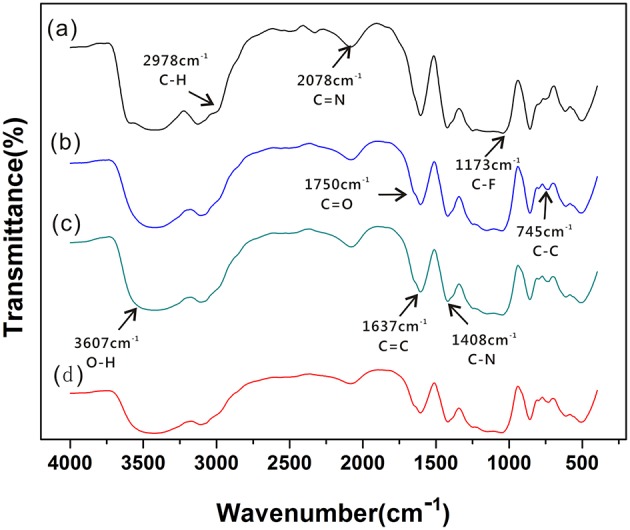
FT-IR spectra of SPE under different NMP content conditions: **(a)** 1:1:3; **(b)** 1:1:4; **(c)** 1:1:5; and **(d)** 1:1:6.

Information on the structural characteristics of SPE was obtained from detailed analysis of the IR region between 200 and 4,200 cm^−1^. Figure 3 shows that different ratios of the prepared SPE have a similar molecular structure. Figures 3a,c show the characteristic bands at 1,637, 2,078, and 1,408 cm^−1^. These are attributed to the stretching vibration of C=C, C=N, and C-N from [EMIM] [BF_4_] (Sergeev et al., 2018). The intensity of C=C and C=N stretching vibration absorption peak decreases with decreasing [EMIM] [BF_4_] concentration. There are characteristic bands at 3,607 cm^−1^ (attributed to stretching vibrations of O-H) and 2,978 cm^−1^ (attributed to stretching vibrations of saturated C-H). In addition, the intensities of the characteristic bands at 1,173 cm^−1^ are associated with stretching vibrations of C-F because of the continuous addition of PVDF during SPE production. Meanwhile, the presence of bands at 1,750 cm^−1^ in Figure 3b are attributed to stretching vibrations of C=O of NMP. The IR data shows that the positions of these absorption peaks did not change much indicating that [EMIM] [BF_4_] and PVDF have good miscibility in NMP, and the crystal structure of PVDF remains constant. The peak due to the β-Phase is identified at 886 cm^−1^ (Ibtisam et al., 2014). With increasing of PVDF concentration, the β-phase intensity of PVDF increases gradually, and the β phase intensity in Figure 3a is obviously larger. This may be an important influencing factor affecting the formation of SPE pore structure and the gas sensitivity of the sensor.

### XPS Analysis of SPE

**Figure 4:**
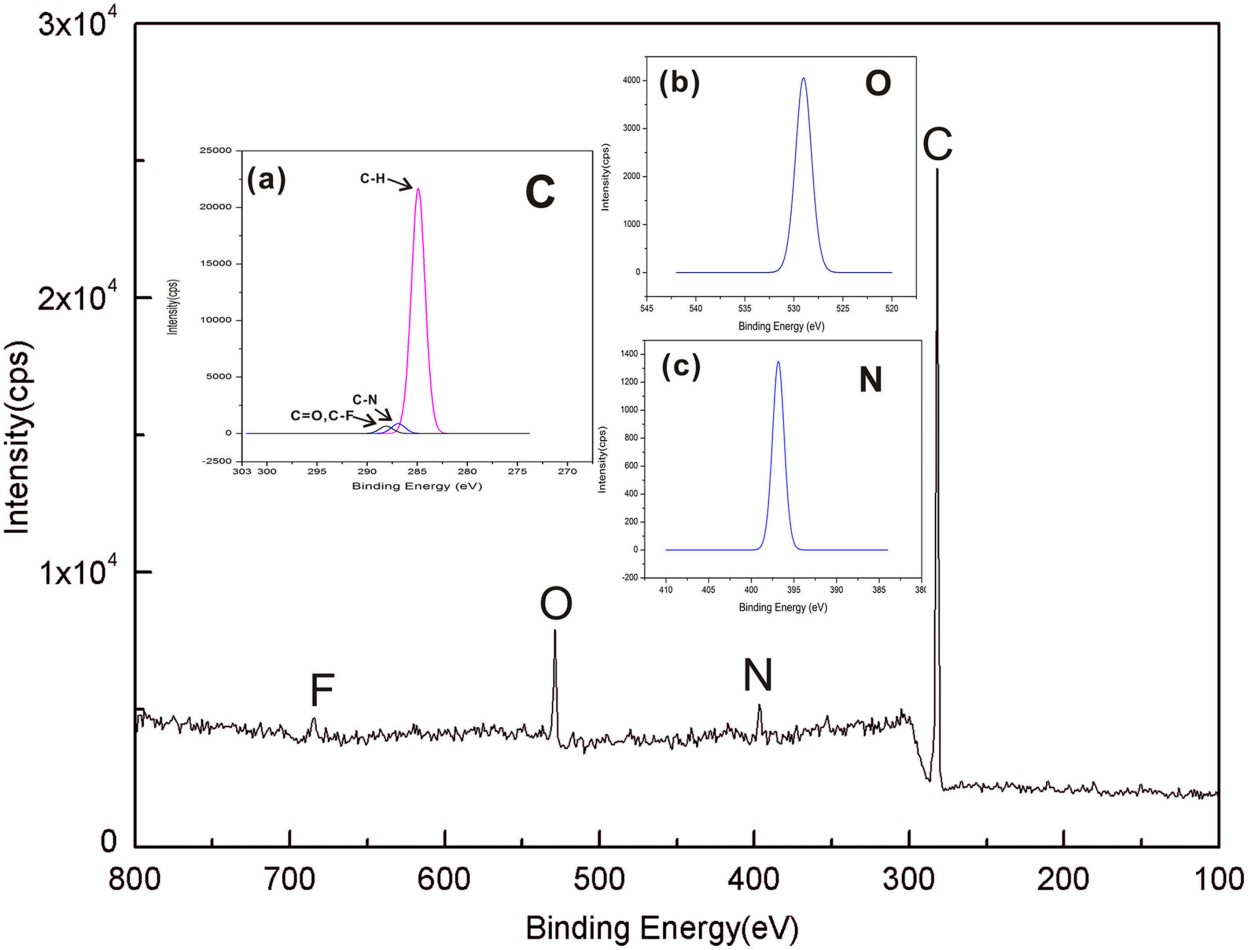
Wide-scan XPS spectra of SPE; **(a)** C1s in SPE; **(b)** O1s in SPE; and **(c)** N1s in SPE.

XPS can investigate the surface elemental compositions and chemical structures of the SPE. The contents of C1s, O1s, F1s, and N1s are shown in Figure 4, and the XPS spectra of C1s, O1s, and N1s are shown in Figures 4a–c. There are four bands corresponding to C atoms, O atoms, N atoms, and F atoms, respectively, and the chemical composition ratio of C: N: O: F in SPE is as follows: 89.29: 3.15: 6.18: 1.38. Figure 4a shows that SPE has three specific peaks at 287.8 eV (C=O, C-F), 286.1 eV (C-N), and 284.7 eV (C-H). These peaks are consistent with the major chemical composition of the constructed SPE. This result confirms that the [EMIM] [BF_4_] mixed with PVDF and did not produce a new binding energy. The SPE has specific peaks, which are seen separately in Figures 4b,c. These peaks confirm the existence of the two binding energies shown in Figure 4a, while further confirming that no new binding energy is produced between the substance that makes up the SPE.

### XRD Analysis of SPE

**Figure 5:**
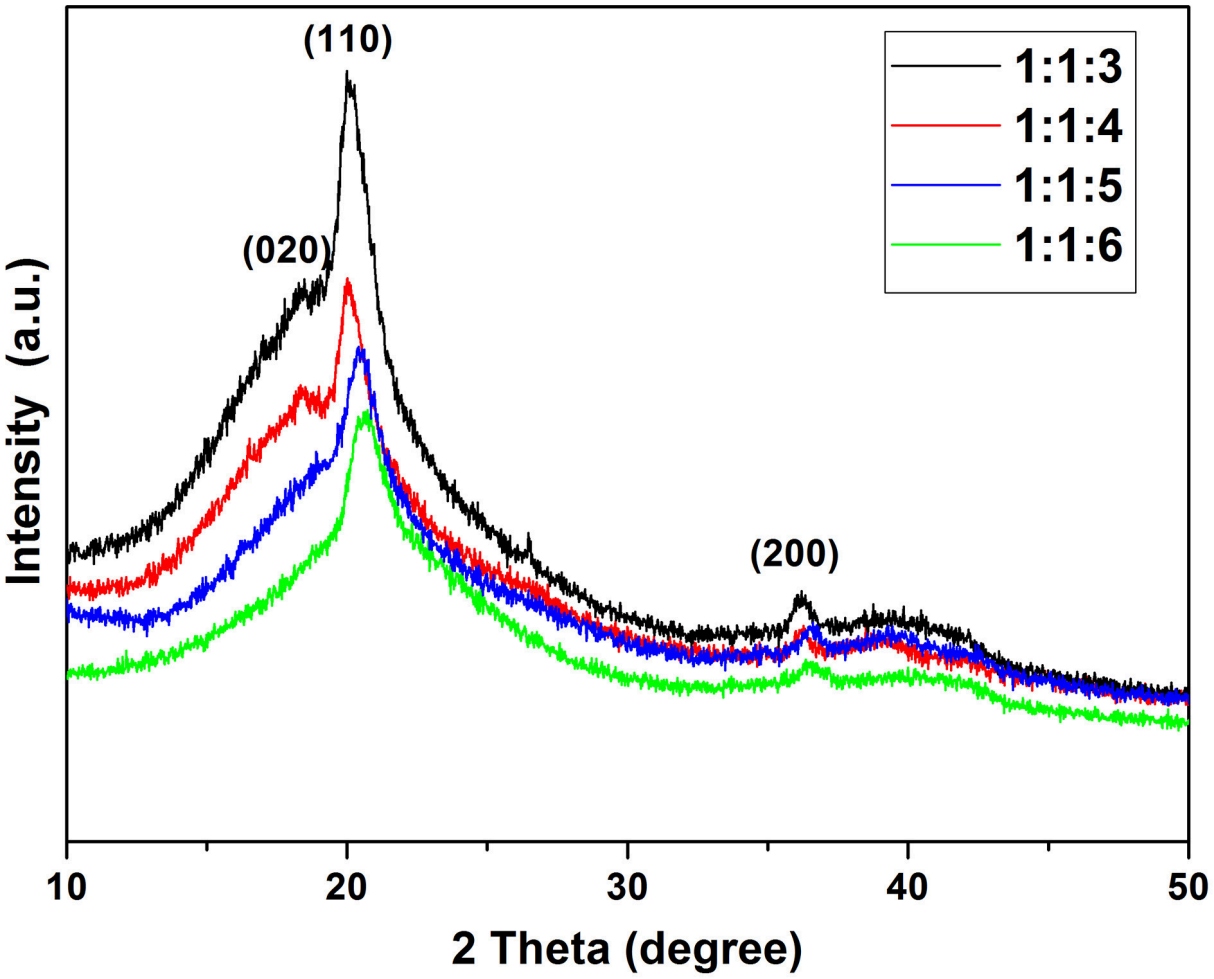
XRD pattern of SPE under different NMP content conditions.

Figure 5 shows the XRD patterns of films of SPE. There are 3 phases in the SPE film, β-Phase (as major phase) at 2θ = 20.3°, α-Phase at 2θ = 18.875°, and γ-Phase at 2θ = 36.1°. These peaks correspond to the diffractions in planes (110), (020), and (200), respectively, all characteristic of the PVDF (Claudia et al., 2010). XRD patterns of different proportions of SPE show that the concentration of NMP in SPE significantly affects the crystallinity of PVDF and the increase of NMP content significantly reduces the β-Phase of PVDF. It can also be seen from the XRD patterns that excessively high NMP concentration will also cause the loss of α-Phase (at ratio of 1:1:6). The effect of NNP concentration on the crystallinity of the SPE sample was also confirmed by FTIR measurements. Versus other samples, the SPE prepared with 1:1:3 exhibits too high crystallinity, which may seriously affect the pore structure of SPE. The reduction of amorphous region will also decrease conductivity and affect gas sensitivity. None of the samples had new crystalline phases indicating that the NMP concentration has no effect on the crystalline phase.

### Effect of the Type of SPE

**Figure 6:**
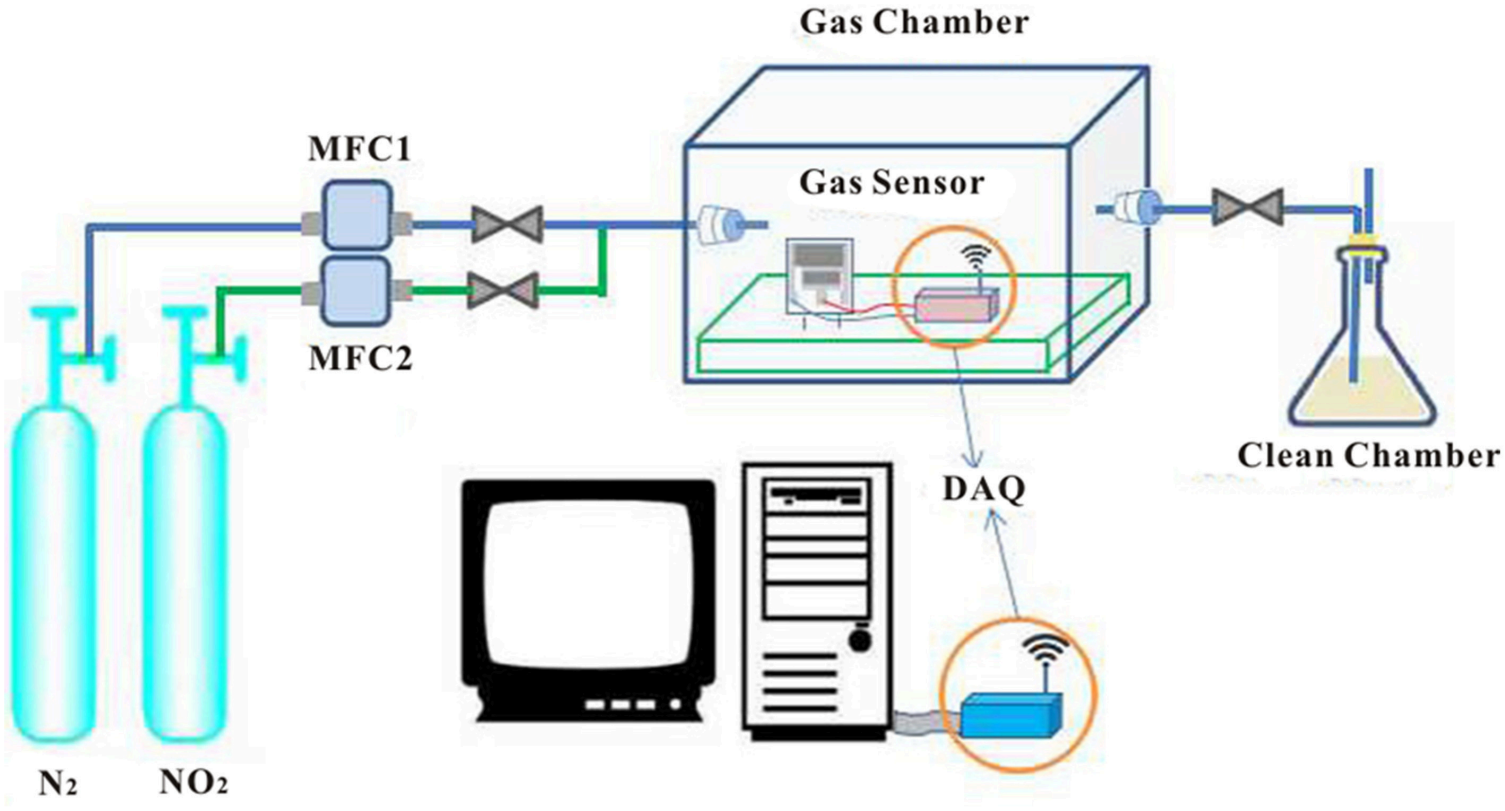
NO_2_ detection process (mixing N_2_ and NO_2_ to reach the test concentration is controlled by two flow controllers, and the gas sensor in the closed reaction chamber detects NO_2_. The sensor detects NO_2_ and generates a change in current, which is transmitted to the computer via wireless sensor technology. The detection system is also equipped with an exhaust gas recovery device).

SPE is an important factor for regular functioning of the sensor. [EMIM] [BF_4_], PVDF, and NMP can form a solid electrolyte with a pore structure. We systematically studied the effect of different proportions and different heat-treated SPE on the gas sensitivity of the sensor. Gas sensitivity experiments were performed on SPE sensors coated with different ratios according to the test flow chart (Figure 6). After the gradual introduction of 175–700 mol of NO_2_, sensor sensing data were acquired and fitted to the calibration curve of the specific sensor to present dependence of sensor current on NO_2_ concentration. The sensitivity was determined as the slope of the calibration curve.

**Figure 7:**
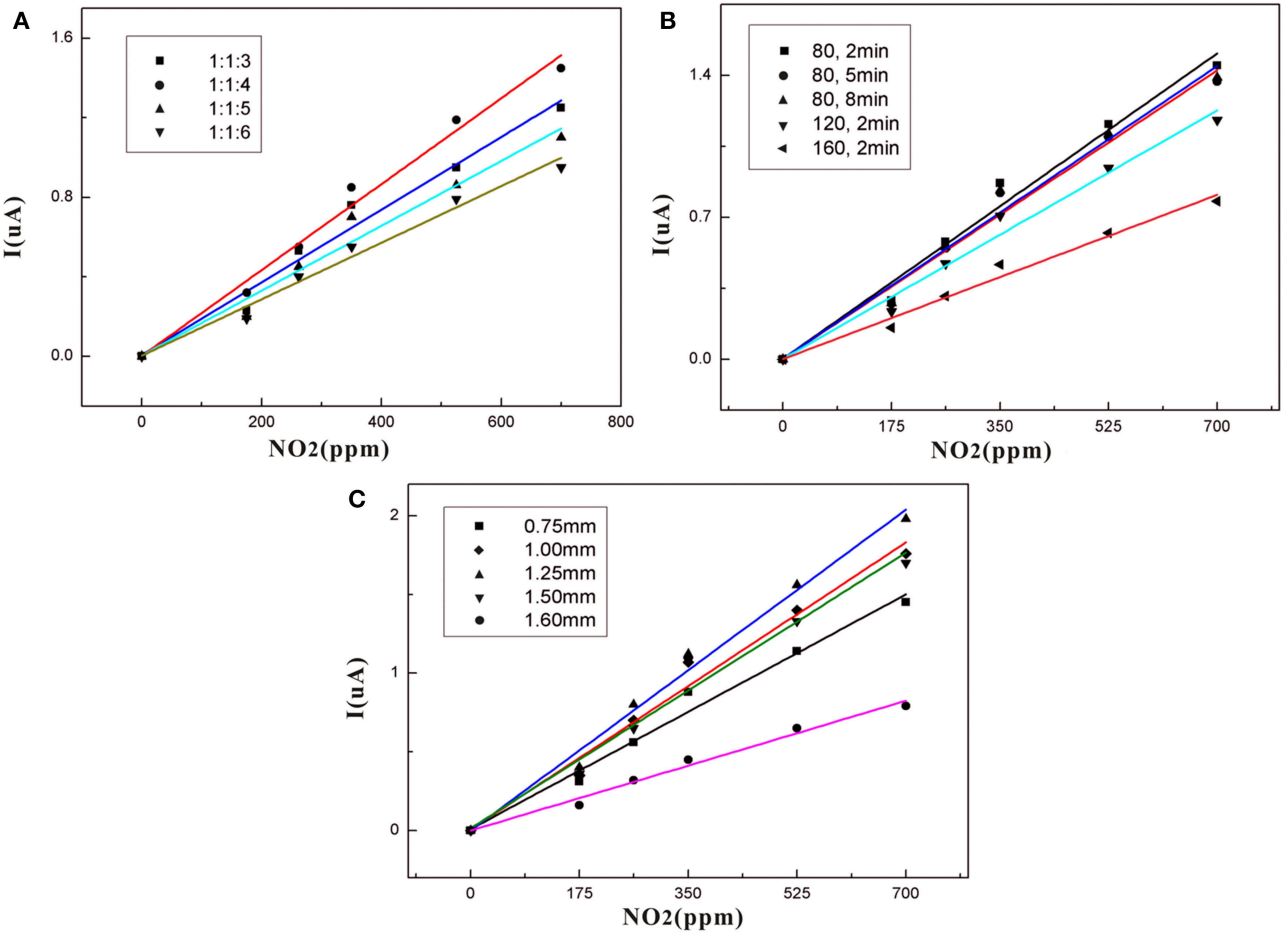
**(A)** Dependence of sensor current on NO_2_ concentration for different NMP contents in the SPE; **(B)** Dependence of sensor current on NO_2_ concentration for different thermal treatments in SPE; **(C)** Dependence of sensor current on NO_2_ concentration for different thicknesses in SPE.

Figure 7A shows that the sensor based on a 1:1:4 ratio of SPE has the best gas sensitivity compared with other samples. This may be because PVDF forms a better pore structure at this ratio. As the concentration of [EMIM] [BF_4_] increases in the low concentration range, free [EMIM]^+^ and [BF_4_]^−^ increase in the SPE, and the solubility of SPE to NO_2_ and conductivity is enhanced. This improves gas sensitivity. However, with further increases in PVDF concentration, the change of PVDF crystallinity may lead to the internal structure of SPE becoming tight with decreasing porosity. This affects the gas sensitivity.

Table 2 shows that as the concentration of the added ionic liquid increases, the conductivity of the SPE without PVDF also increases. However, this is not true for the relationship between sensor sensitivity and the conductance of the SPE. Table 3 shows that the SPE prepared at a ratio of 1:1:4 has the highest conductivity. This may be because the concentration of PVDF is too high and results in excessive SPE crystallinity with too high of a viscosity; thus, the amorphous region of SPE is reduced, and the decrease in ion mobility leads to a decrease in electrical conductivity. The difference in sensor sensitivity shown in Figure 7A indicates that the conductivity of the SPE also plays an important role in the sensitivity of the NO_2_ sensor.

**Table 2:** Conductance values of mixed [EMIM] [BF_4_] and NMP.

**[EMIM] [BF4]: NMP**	**1:3**	**1:4**	**1:5**	**1:6**
AC conductance (ms/cm)	18.82	17.35	13.47	10.25

**Table 3:** Conductance values of the SPE.

**PVDF: [EMIM] [BF4]: NMP**	**1:1:3**	**1:1:4**	**1:1:5**	**1:1:6**
AC conductance (ms/cm)	3.76	4.62	1.31	0.8

The heat treatment time of the SPE at 80°C does not impact the gas sensitivity of the sensor (Figure 7B). Importantly, the heat treatment temperature is increased, and the gas sensitivity is lowered. This means that an increase in the heat treatment temperature may change the crystalline form of PVDF, which may affect gas sensitivity. The SPE based on the best gas-sensitivity ratio and heat treatment temperature was selected for further detailed study because it exhibited the highest sensitivity. This was coated on electrodes in five thicknesses. The five gas sensitivity curves (Figure 7C) indicate that the SPE has the best gas sensitivity at a sensor thickness of 1.25 mm. As the thickness increases, it becomes harder for the NO_2_ involved in the redox reaction to come into contact with the electrode surface. However, the SPE volume is reduced when the thickness of the coating is <1.25 mm resulting in conductivity and gas solubility that are incompatible with normal sensor requirements. The sensor calibration fit curve in Figure 7 is normalized for comparison. Here, *I* represents the current response of the sensor while analyzing the exposure.

**Figure 8:**
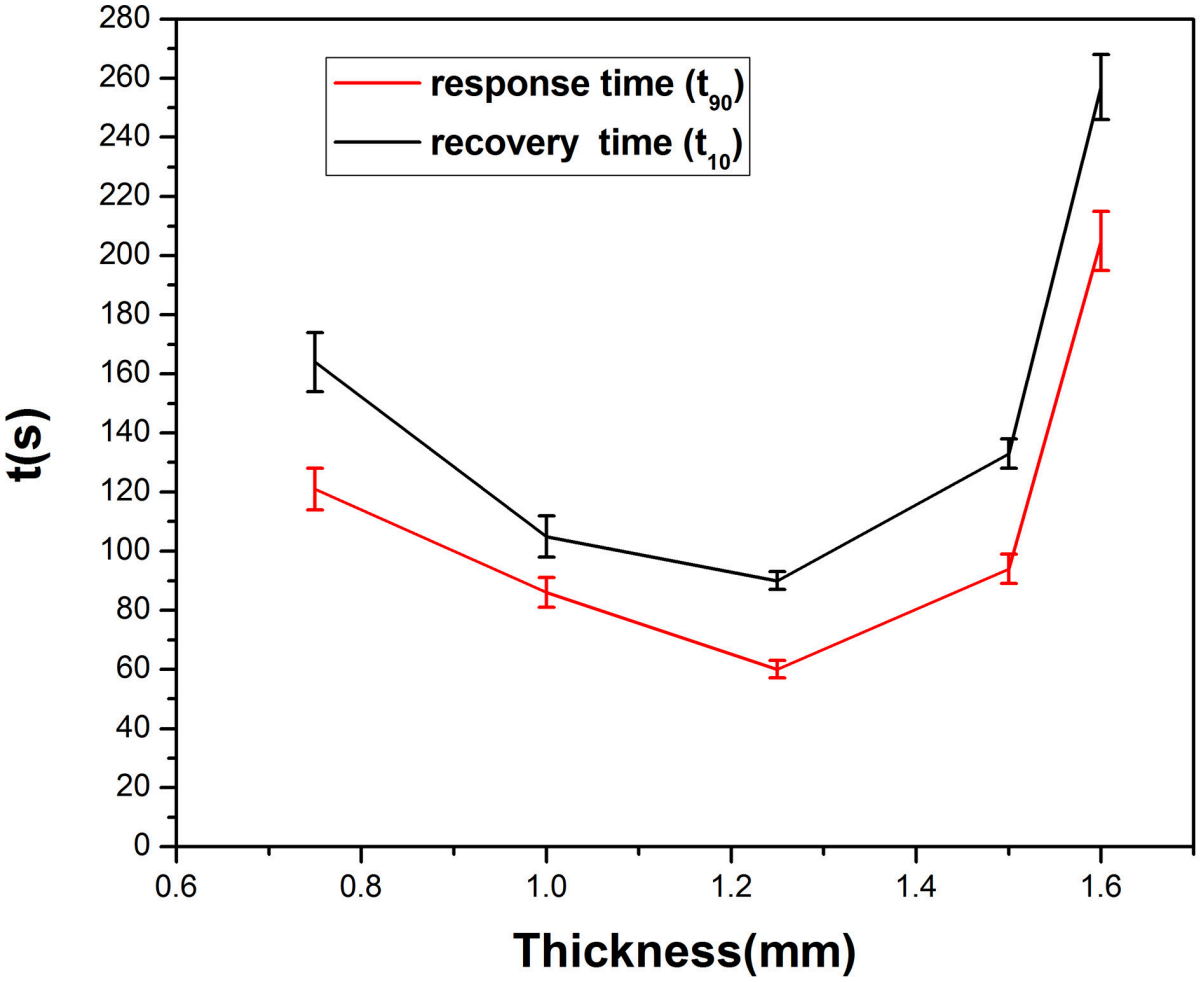
Response time of different SPE thicknesses.

Figure 8 gives information on the relationship between response times (rise time and recovery time) and the thickness of the SPE. The rise/recovery time (t_90_/t_10_) was determined as the time period required to achieve 90 or 10% of the full response current upon a step increase/decrease in NO_2_ exposure (Mason et al., 1994; Webb et al., 2012; Kuberský et al., 2013). There is longer response time when the thickness is over 1.25 mm and below 1.25 mm. This result indicates that NO_2_ detection is strongly dependent on the SPE thickness. The suspected cause of this dependence is the degree of contact of NO_2_ with the electrode and the conductivity of the SPE.

### Morphology and Microstructure of SPE

**Figure 9:**
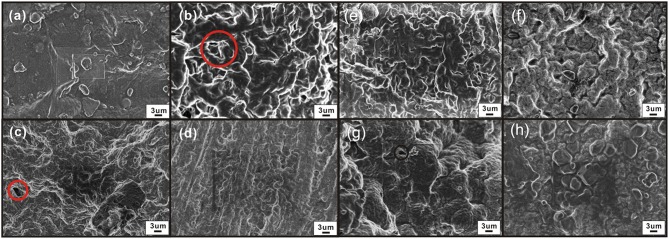
Morphology of the SPE layer under different NMP contents, thermal treatment times, and drying temperature conditions (electron microscope, magnification 15,000×): **(a)** 1:1:3 weight ratio of an [EMIM] [BF_4_], PVDF, and NMP, **(b)** 1:1:4; **(c)** 1:1:5; **(d)** 1:1:6; **(e)** 80°C for 5 min; **(f)** 80°C for 8 min.; **(g)** 120°C for 2 min; and **(h)** 160°C for 2 min.

We analyzed the morphology and microstructure of SPE to verify the difference in gas sensitivity. The SPE layer has different surface morphologies under different NMP ratios (Figures 9a–d). The SPE layer with the least amount of NMP (Figure 9a) is a compact plane with almost no pore mechanism indicating that PVDF and [EMIM] [BF_4_] account for a larger proportion; it is difficult to form pore structures. In contrast, the SPE layer with the most NMP content (Figure 9d) is relatively loose. It has smaller pores, and the effective surface of both is less than the labeled portions in Figures 7B,C. Figure 9b shows that the SPE layer consists of [EMIM] [BF_4_], PVDF, and NMP at a weight ratio of 1:1:4. This contains a significantly larger pore structure, which helps the interdigitated electrode to make better contact with NO_2_. This confirms that the sensitivity difference shown in Figure 7A is closely related to the pore structure.

As shown in Figures 9a–f, the pore structure of the SPE is obvious on the surface of the SPE treated at 80°C for different times. The void ratio is similar. This indicates that the drying time has no significant effect on the formation of the pore structure of the SPE layer and does not affect the dispersion and crystallization of PVDF in the NMP. This verifies that the SPE sensors prepared at different drying times have similar gas sensitivity results (Figure 7B).

Figures 9a,g,h are from the surface of the SPE layer: the pores are smaller and fewer in number when the temperature rises. The surface of the SPE is almost free of pores, and the surface is denser and smoother at 160°C. This may arise from the higher crystallization temperature causing excessive deformation and crowding of PVDF during heat treatment. As a result, the cross-linking capacity is destroyed, and the porosity is reduced—this affects the gas sensitivity of the sensor.

### Gas Sensor Principle

The principle underlying this SPE-based amperometric NO_2_ sensor is the measurement of the current produced by the electrochemical reduction of NO_2_ on the electrode. NO_2_ contacts the SPE and reaches the working electrode through the pores in the SPE followed by electrochemical reduction. At the same time, the ionic liquid contained in the SPE has a certain gas solubility—this promotes the reduction of NO_2_ on the working electrode (Buzzeo et al., 2004). The generally accepted NO_2_ reduction reaction at work electrode is represented by Equations (1) and (2).

(1) $$NO_{2}+e^{-}\to NO_{2}^{-}$$

(2) $$NO_{2}^{-}+2H^{+}+e^{-}\to NO+H_{2}O$$

A more definite description of the reduction mechanism would require a specialized study involving identification and quantification of the reaction products. Most probably, water is oxidized at the auxiliary electrode:

(3) $$H_{2}O\to 2H^{+}+2e^{-}+1/2O_{2}$$

Thus, the overall reaction occurring in the sensor can be expressed as

(4) $$NO_{2}\to NO+1/2O_{2}$$

**Figure 10:**
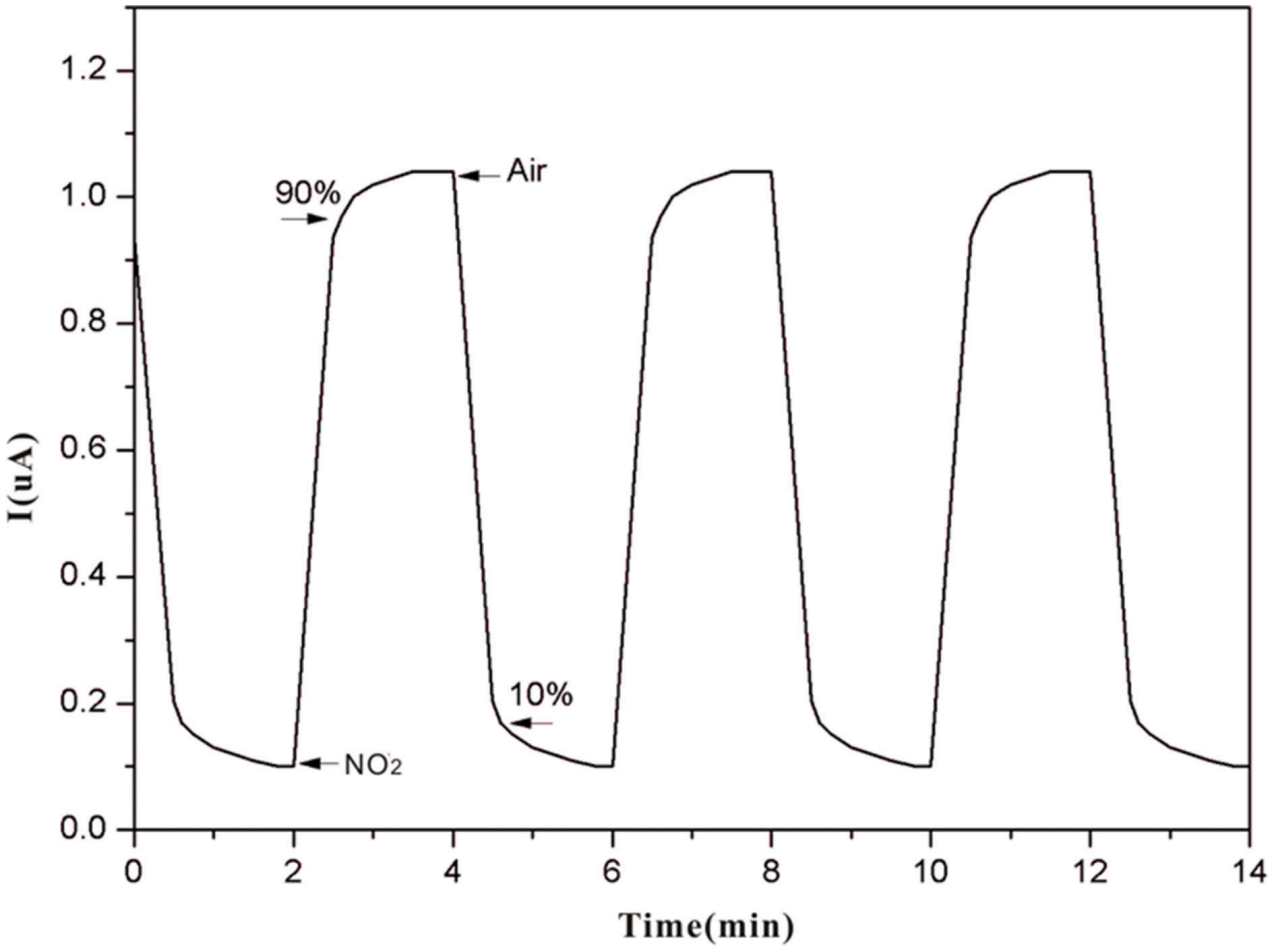
Sensor response to repeated exposures to 100 ppm NO_2_.

The current through the sensor is controlled by the reduction reaction of NO_2_ provided that the oxidation reaction of the water in the reaction produces protons and the proton transfer rate from the auxiliary electrode to the working electrode is sufficiently high. The sensor composition remains unchanged during the NO_2_ detection. As shown in Figure 10, the sensor response has excellent stability and reproducibility with fast response/recovery times (60/90 s).

## Conclusion

We report a systematic study of an electrochemical NO_2_ sensor with an ionic liquid-based SPE and screen-printed carbon electrodes. The sensor gas sensitivity data showed that a 1:1:4 weight ratio [EMIM] [BF_4_], PVDF, and NMP had the highest sensitivity. These were dried at 80°C for 2 min and coated on the PET film at a thickness of 1.25 mm. The results demonstrate that the two-layer sensing structure of the interdigital electrode and the SPE can reliably and reproducibly respond to NO_2_. FTIR and XPS proved that the synthesis mode of SPE is physical miscibility. Combined with the sensitivity analysis, the effect of SPE on the NO_2_ sensor is inseparable from the crystal morphology of PVDF. Moreover, the SEM and XRD of the SPE layer shows that the sensitivity of the sensor strongly depends on the interconnected pore structure formed by PVDF crystallization in SPE. A lower proportion of NMP will lead to a lower formation of pores. On the contrary, an excessive proportion of NMP causes the pore dispersion of PVDF in SPE to become smaller. The crowding of the PVDF substrate is increased with higher heat treatment temperature and longer heating time. In addition, gas sensitivity is also related to the thickness and conductivity of SPE. SPE containing room temperature ionic liquids expands the range of applications of solid electrolytes. The solid electrolyte prepared here can be used to design gas sensors operating at room temperature, and can be used as an alternative to the existing NO_2_ sensor in future research and development.

## Author Contributions

RL, BD, and SZ contributed to the conception and design of the study. YC contributed to synthesis and analyses. HL contributed to manuscript writing and revision. All authors made substantial, direct, and intellectual contributions to the work, and approved it for publication.

### Conflict of Interest Statement

The authors declare that the research was conducted in the absence of any commercial or financial relationships that could be construed as a potential conflict of interest.
